# Ornithophily in the subtribe Maxillariinae (Orchidaceae) proven with a case study of *Ornithidium fulgens* in Guatemala

**DOI:** 10.1038/s41598-022-09146-4

**Published:** 2022-04-04

**Authors:** Monika M. Lipińska, Fredy L. Archila, Łukasz P. Haliński, Dorota Łuszczek, Dariusz L. Szlachetko, Agnieszka K. Kowalkowska

**Affiliations:** 1 Department of Plant Taxonomy and Nature Conservation, Faculty of Biology, University of Gdańsk, Wita Stwosza 59, 80-308 Gdańsk, Poland; 2 Foundation Polish Orchid Association, Abrahama 15A/2, 81-825 Sopot, Poland; 3 Estación Experimental de Orquídeas de la Familia Archila, 1a Av. 5-28, Zona 1, 16001 Cobán A.V., Guatemala; 4 Herbario BIGU, Escuela de Biología, Facultad de Ciencias Químicas y Farmacia, Universidad de San Carlos de Guatemala, 01012 Guatemala City, Guatemala; 5 Department of Environmental Analytics, Faculty of Chemistry, University of Gdańsk, Wita Stwosza 63, 80-308 Gdańsk, Poland; 6 Laboratory of Electron Microscopy, Faculty of Biology, University of Gdańsk, Wita Stwosza 59, 80-308 Gdańsk, Poland; 7 Department of Plant Cytology and Embryology, Faculty of Biology, University of Gdańsk, Wita Stwosza 59, 80-308 Gdańsk, Poland

**Keywords:** Plant cell biology, Plant ecology

## Abstract

Ornithophily has been long speculated to occur in the subtribe Maxillariinae (Orchidaceae), relying either solely on micromorphological analyses or scarce field observations of undefined species. In Guatemala we were able to observe regular visits of the azure-crowned hummingbirds feeding on flowers of *Ornithidium fulgens*. These observations have led us to investigation of floral attractants by means of scanning and transmission microscopy, histochemical and chemical analyses (GC–MS). Conducted investigation revealed that the epidermis of basal protuberance of column-foot has features proving the secretory activity and that secreted nectar is sucrose-dominant. Slight secretion on the middle part of the lip is puzzling. The presence of other potential pollinators has not been reported. Based on the results of this study, we confirmed that the flowers of *O. fulgens* meet all criteria of ornithophily and thus that the hypothesis about bird pollination in the subtribe Maxillariinae is proven. The presented results confirm that the previously described floral features predicting the bird pollination in this group are justified. This strengthens the theory about floral adaptations to different pollinators and gives valid reasons to consider species with flowers with a certain set of traits as ornithophilous, even in the absence of the pollination observation.

## Introduction

Orchids are well known as one of the most advanced groups of plants in terms of adaptation to different forms of animal pollination. Ornithophily (bird pollination) has evolved several times in many plant groups, usually by deriving from bee pollination. It is particularly widespread in tropical and subtropical areas with constant availability of nectar-rich flowers, which provide a food reserve for nectarivore birds. In regions where vegetation has a long dormant period, bird pollination usually does not occur or is occasional. North America is an exception, as hummingbirds migrate north during the summer^1^. There are some features that make birds great pollinators, and these are for example long flight distances and high visual acuity. Their role is especially important when unfavorable weather conditions are causing a decrease in the activity of other pollinators, such as bees^1^. In environments where the populations of insects are not abundant, such as for example high-altitude ecosystems, birds may thus constitute an important group of pollinators^2^.

Specific features such as flower morphology, color, nectar production, and odor presence determine the suitability for pollination among different groups of animals^3–7^. Although the complexity of the pollination systems is usually higher than floral morphology initially would suggest^8^. There is some evidence supporting a strong association between certain floral traits and functional groups of pollinators that exert similar selective pressures^5^. The main pollinators of Maxillariinae representatives are stingless bees (Meliponini)^9,10^, however, visits of bees from the subtribe Euglossini and bumblebees *Bombus volucelloides* Rolfe have also been observed^2^. It is therefore understandable that bee pollination syndrome is the most common among Maxillariinae. Flowers pollinated by bees are characterised by diurnal anthesis. They are zygomorphic with a prominent landing platform, horizontal, colored in blue, violet, purple, yellow or white. Nectar guides are usually present and complex. The scent is fresh and sweet. Nectar is more or less hidden, in shallow or rather deep containers^2^. Bird pollination, or ornithophily, is a well-recognized syndrome of floral traits. Ornithophilous flowers are often red with copious dilute nectar. Furthermore, they lack characters associated with other pollination syndromes, such as scent ^1^. According to Grant & Grant^11^, floral adaptations to bird pollination can be classified in four broad types: attraction mechanisms, exclusion mechanisms, protection mechanisms, and pollination mechanisms. Attraction mechanisms cover features such as copious nectar production and vivid floral display that attract birds to flowers. The flower color may be simply red or orange, or a combination of contrasting colours, including orange, yellow, green, and blue. The reason for the remarkably consistent association of bird-pollination with red or reddish flowers can be associated with either avoidance of bees and other insect pollinators (as they cannot see these color range) or attraction of birds (red flowers acting as a signal of high caloric reward^1^ and references therein). Exclusion mechanisms are those that help to discourage undesirable flower visitors that might otherwise interfere with pollination and rob nectar. These may be for instance red colour, long and narrow floral tubes, and the absence of insect landing platforms^1^. Birds are rather large and potentially destructive pollinators, thus protection mechanisms seem to be of great importance. These may be for example mechanical strengthening of the flower (sclerenchyma or collenchyma tissue in various floral parts), protection of ovary with ovules by separate localization of ovary and nectary in flowers, sheathing of the ovary by a staminal tube. Alternatively, there may be a groove formed by the corolla to guide birds’ beaks to the nectary without causing damage, or ridges of the corolla to provide direct protection to the ovary^1^. Pollination mechanisms are those that enhance the precise deposition of pollen on beak and stigma. These include both spatial and temporal relations of the reproductive organs to the position of pollinating birds^1^.

Hummingbirds (Trochilidae), the sunbirds (Nectariniidae), and the honey-eaters (Meliphagidae) are thought to be the major pollinators. The distribution range of the first ones is limited to the New World, and ranges from southern South America to Alaska, reaching the highest diversity in the northern Andes^11^. The major evolutionary radiation of this group has happened in South America, whereas the secondary radiation occurred in North America^12^. However, as fossil evidence found in Europe suggests, the early evolution of hummingbirds was not exclusive to the New World^12^. The hovering behavior is typical for hummingbirds. They are collecting nectar without landing on the plant, which may therefore have hanging or pendant flowers. Small body size compared to non-nectarivorous birds, long and/or curved beaks and extendible tongue with grooved tip are adaptations for feeding the nectar^13,14^, although insects are an important protein source for them^15^. Some species of hummingbirds, such as those from the genus *Amazilia*, are known to hold and defend their territories against intruders and depending on the resource value, thus their pollination behavior may favor self-pollination^13,16–20^.

Maxillariinae Benth. is an exclusively Neotropical orchid subtribe, which embraces about 900 species classified in 14–36 genera^21^. Until now, there was no conclusive evidence for ornithophily in this extremely diverse taxon. Van der Pijl & Dodson^2^ have observed the fiery-throated hummingbird (*Panterpe insignis*) visiting an unidentified species of *Maxillaria* with pink, tubular flowers. Dziedzioch et al.^22^ during the field study on the hummingbird-plant community (assemblage of plants with hummingbirds as principal pollinators) of a tropical montane rain forest in Southern Ecuador have reported visits of *Ocreatus underwoodii peruanus* and *Heliangelus amethysticollis* to six different species of *Maxillaria sensu lato*, however, only two of them were identified to the species level (namely *Ornithidium aureum* Poepp. & Endl. and *O. jamesonii* Rchb. f.). Potential bird pollination in these species is not supported by any micromorphological analysis and there is also no evidence whether these visits were simply accidental or intentional and resulted in fruit production. On the other hand, Stpiczyńska et al.^23,24^ have conducted micromorphological analysis of red flowered *Ornithidium coccineum* (Jacq.) Salisb. *ex* R. Br. (as *Maxillaria coccinea* (Jacq.) L.O. Williams *ex* Hodge) and *O. sophronitis* Rchb. f., and concluded that in terms of morphology, they meet a range of criteria characteristic of the hummingbird-pollinated flowers, but differ from those in the presence of sweet honey scent. However, these studies are lacking the support of actual field observations. Nevertheless, it is worth mentioning that the detailed results from the analysis of floral morphology and anatomy may suggest certain pollination syndrome and give the insight into specific pollination mechanisms, such as Darwin’s predictions of *Angraecum sesquipedale* being pollinated by the long tongued moth, described later as *Xanthopan morganii praedicta* [and references therein^25^].

The main aim of the presented research was to test long suspected ornithophily in the subtribe Maxillariinae on the example of *Ornithidium fulgens *Rchb.f. (= *Maxillaria fulgens* (Rchb.f.) L.O.  Williams). This paper emphasises the evidence for ornithophilous syndrome by means of macromorphological, micromorphological, anatomical, histochemical, ultrastructural and chemical analysis. Moreover, the photographs of the hummingbirds approaching the flowers and transferring the pollinia are registered.

## Results

### Field observations

**Figure 1:**
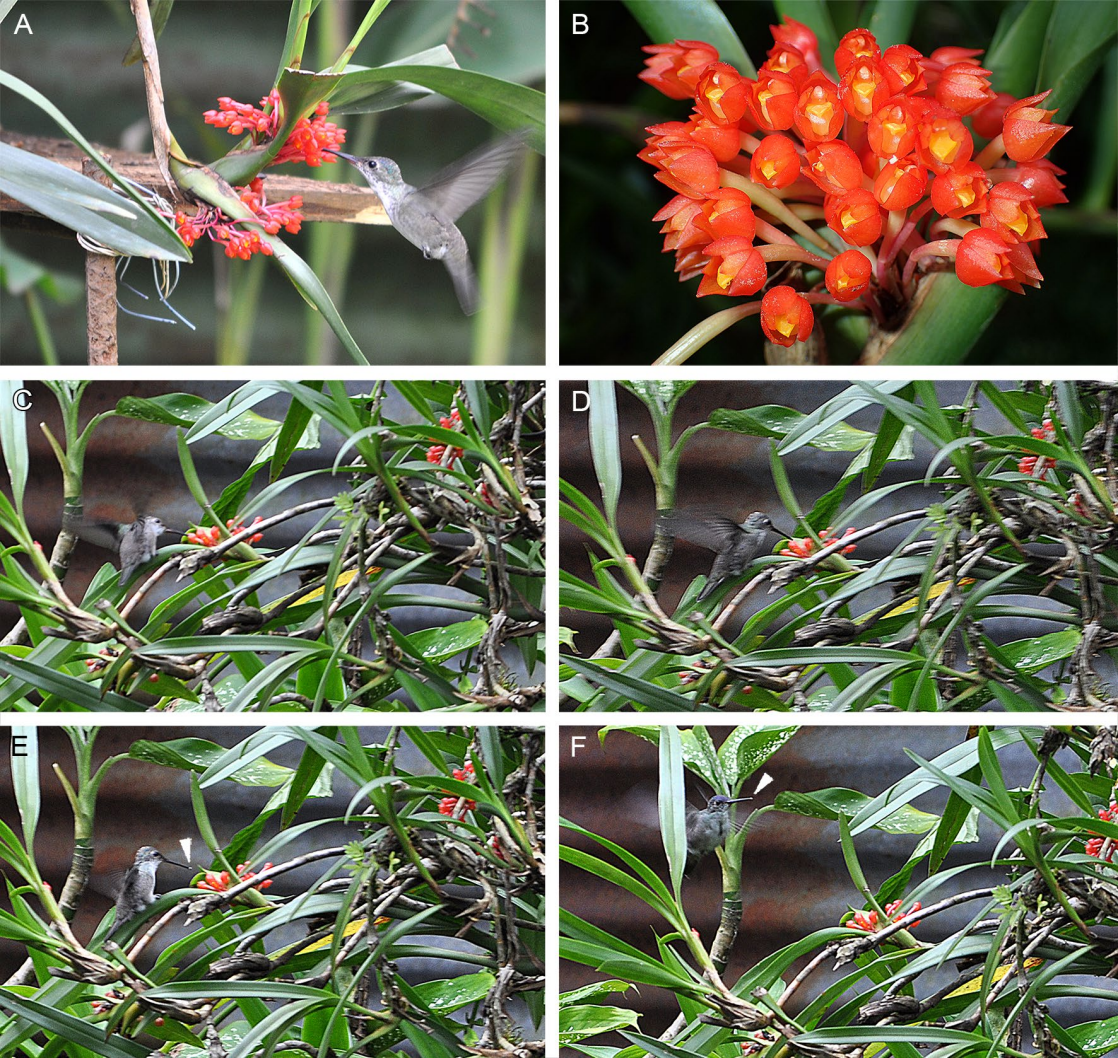
Observations in Estación Experimental de Orquídeas de la Familia Archila (Cobán, Guatemala) in 2017–2020: (**A**) The azure-crowned hummingbirds (*Amazilia cyanocephala*) regularly visiting the flowers of *Ornithidium fulgens*; (**B**) Inflorescence: flowers with predominating red colour and contrasting yellow lip; (**C**–**F**) Sequence of photos showing the moments of the hummingbird's visit, which results in the collection of pollinia; (**E**, **F**): pollinia attached to the beak (*arrows*). Phot. A, C-F: F. Archila; B: M. Lipińska.

During field studies conducted in Estación Experimental de Orquídeas de la Familia Archila (Cobán, Guatemala) in 2017–2020, we have witnessed regular visits of the azure-crowned hummingbirds (*Amazilia cyanocephala*) feeding on flowers of *O. fulgens* (Fig. 1A–F). In general, ornithophily has been frequently recorded by the second co-author during the past 20 years, both in the station and forest. During these long-time observations, pollinia transport has been spotted. While conducting an investigation focused only on documenting this phenomenon, we have run the observations for one week, in the early mornings and later in the day, from midday until sundown. Such observations are valuable, however limited, thus they require continuation in the future. *Amazilia cyanocephala* was the only species that has ever been spotted by us while transferring the pollinia on the beak (Fig. 1A,C–F). Thus, it remains unknown if other hummingbird species may also serve as pollinators to *O. fulgens*. A possible reason for that is the fact that representatives of the genus *Amazilia* are known to be territorial. In the Station, about 10–15 individuals of *O. fulgens* are growing and while visiting, the hummingbird approached about 10 flowers each time. After the visit, the flowers were being marked to enable tracking the fruit set, which can be estimated at about 80–90%. What is worth mentioning, in the absence of the hummingbirds, the fruit set has not been recorded. The presence of other potential pollinators has not been reported.

### Macromorphology

Plants are robust epiphytes bearing pseudobulbs and leaves separated by elongate rhizome segments. Stems are terete, woody, about 7 mm in diameter. Pseudobulbs are ovoid in general outline, subtended and largely hidden by 2–5 foliaceous bracts with blades indistinguishable from the leaves, and separated by 10–30 cm long rhizome segments. Leaves are lanceolate, acute. Inflorescences (Fig. 1B) are subsessile, forming fascicles of 2–8 flowers, with peduncle 2–3 cm long and minute floral bracts. Flowers are numerous, globose, red with a yellow lip, rigid, pendant, without detectable fragrance. Sepals are broadly elliptic-ovate, obtuse, deeply concave. Lateral sepals are oblique. Petals are oblong-elliptic, obtuse. Lip is essentially unlobed, sigmoid in longitudinal section, very fleshy, rigidly attached to the column, ca. 5 mm long in natural position, saccate, without callus, limb ovate, obtuse, very fleshy. Lip margins are curved. Gynostemium is short, stout, about 3 mm long, with a swollen mound at the base and indistinct foot. Pedicel and ovary are ca.1–1.5 cm long.

### Micromorphology

**Figure 2:**
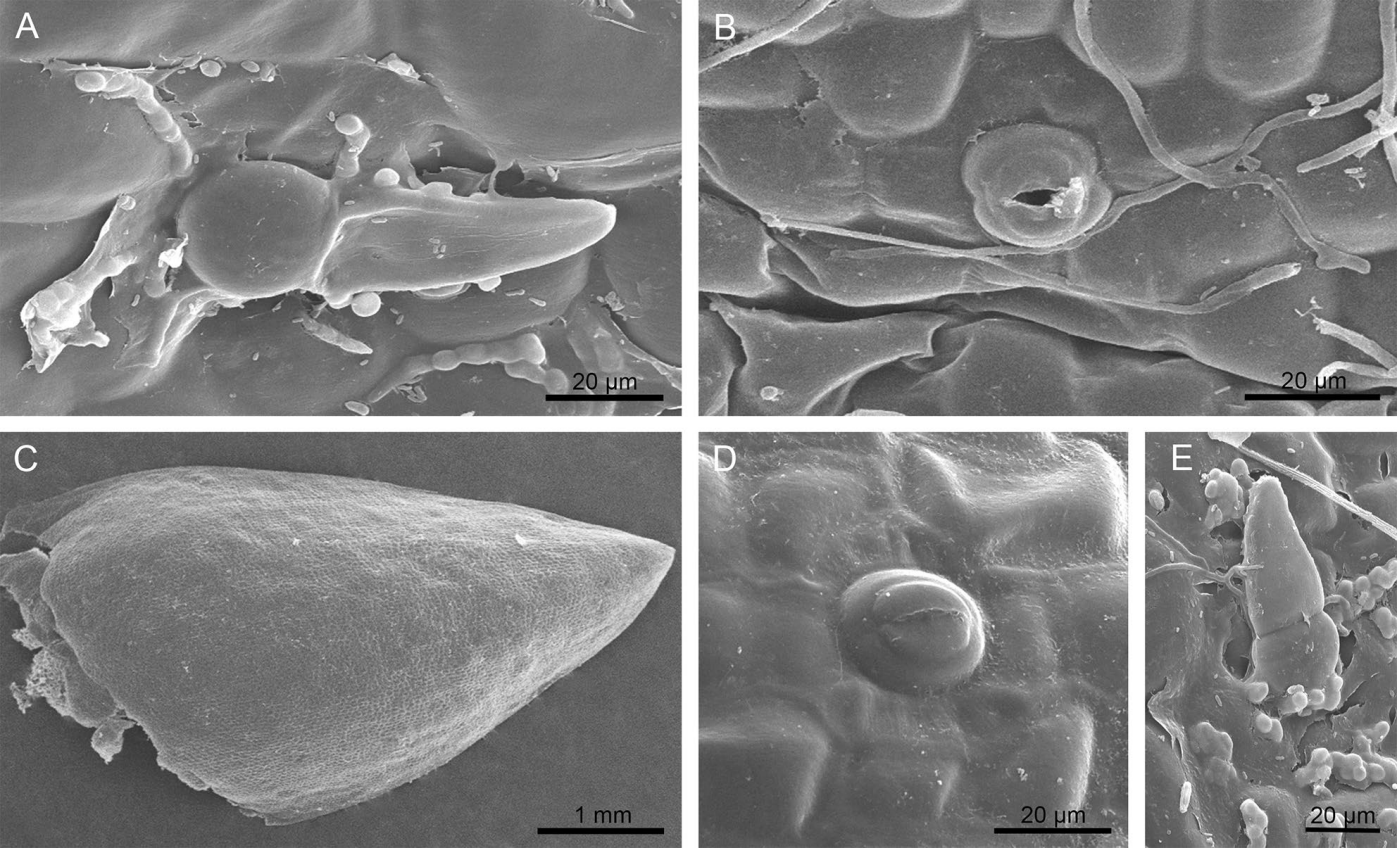
Micromorphological studies (SEM): (**A**) Dorsal sepal: two-celled trichomes scattered on the outer (upper, abaxial) surface, covered by remnants of secretions. Fungal spores and hyphae were noticed on the surface; (**B**) Dorsal sepal: the glabrous inner (lower, adaxial) surface at the apex with a paracytic type of stomata. Some fungal hyphae also present on the surface; (**C**) Lateral sepal: glabrous outer surface with some secretory residues; (**D**) Lateral sepal: glabrous outer surface with paracytic type of stomata; (**E**) Lateral sepal: the two-celled trichomes with secretory residues on the outer surface. Fungal spores and hyphae were also noticed.

**Figure 3:**
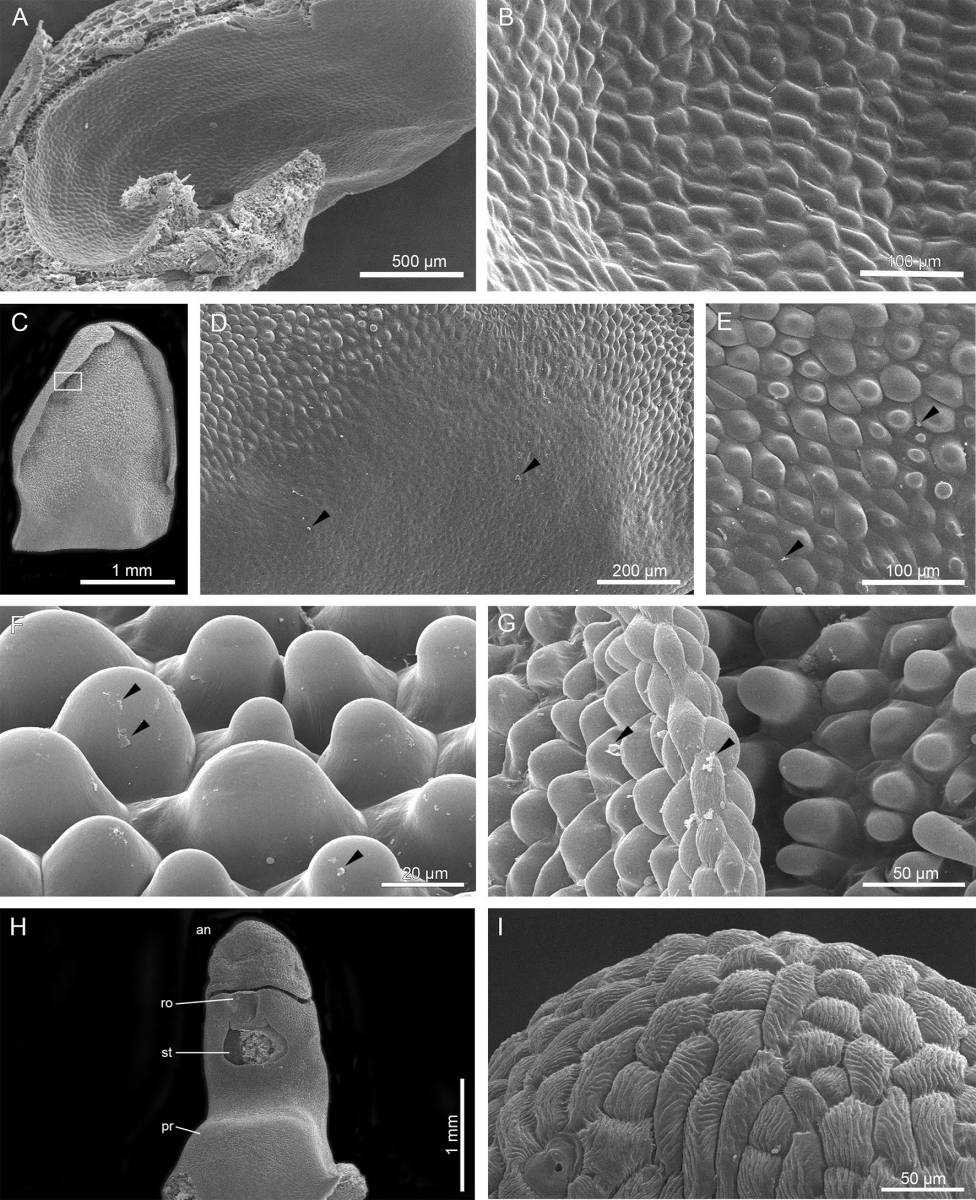
Micromorphological features of the lip (SEM): (**A**) Lip: the inner surface of the saccate base; (**B**) The inner surface—details of A; (**C**) Lip: the glabrous middle part passing into a papillate, distal part—densely papillose with conical and obpyriform papillae and curved margins; (**D**, **E**) Details of the middle part showing the passage from glabrous to papillose surface, remnants of secretions (*arrows*); (**F**) The distal part with obpyriform papillae with residues of secreted material (*arrows*); (**G**) Lip margin and papillae at distal part, with residues of secreted material (*arrows*), detail of (**C**); SEM studies of gynostemium. (**H**) The stout gynostemium equipped with a swollen mound/protuberance (*pr*) at the base, *an*—anther cap, *ro*—rostellum, *st*—stigma; (**I**) Anther cap glabrous, with a strongly striate cuticle and visible stomata.

Dorsal sepal is glabrous with scattered sessile one or two-celled trichomes, covered by remnants of secretions (Fig. 2A) on the outer side (upper, abaxial surface). Paracytic stomata (Fig. 2B) are present on both surfaces. On the inner (lower, adaxial) surface at the base they are embedded in slight depressions. Lateral sepals are glabrous (Fig. 2C) with paracytic stomata (Fig. 2D) and mainly two-celled trichomes with secretory residues on the outer side (Fig. 2E). Also, two lateral petals, forming inner whorl, are glabrous with paracytic stomata at apices and two-celled trichomes at bases on both sides, and a little visible residue of secreted material. The inner surface of the saccate base of the lip is glabrous (Fig. 3A, B), in the middle part the surface becomes papillate (Fig. 3C–E) and at the distal part is densely papillose with conical and obpyriform papillae (Fig. 3C, F, G) and visible residues of secreted material (Fig. 3E, G). The stout gynostemium is equipped with a swollen mound/protuberance at the base (Fig. 3H). Anther cap is glabrous, with a strongly striate cuticle and visible stomata (Fig. 3H, I). The SEM studies allowed us to describe features of epidermis and select specific floral parts with possible secretory activity for histological tests and TEM studies.

### Histochemistry and ultrastructure

**Figure 4:**
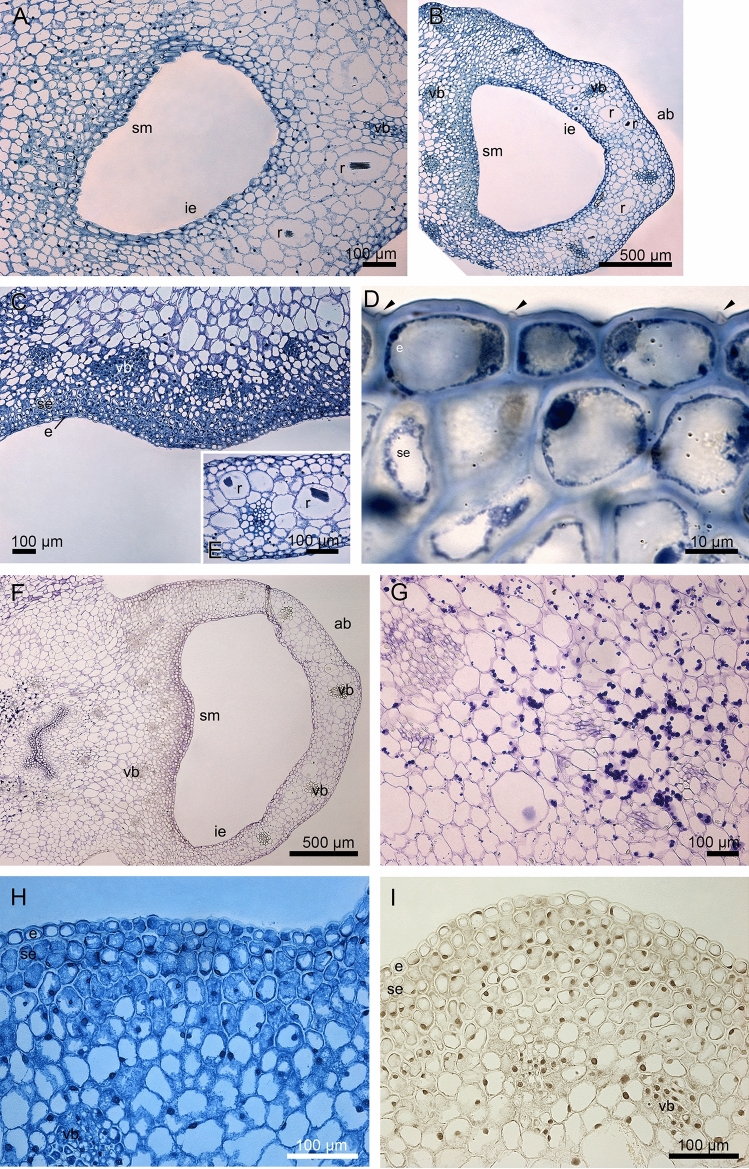
Histochemical tests of the lip and the swollen mound of gynostemium: (**A**–**C**) The transverse sections from the saccate lip base and the swollen mound with dense cytoplasm, only exclusively in the swollen mound of gynostemium, the vacuolized parenchyma cells, collateral vascular bundles (TBO); (**D**) The cuticular swellings noticeable on epidermal cells of the mound (TBO; *arrows*); (**E**) The idioblasts with bundles of raphides—sharp needle-like crystals of calcium oxalate (TBO); (**F**, **G**) the starch grains in deeper layers of parenchyma, not in epidermis (PAS); (**H**) The test for presence of proteins of a swollen mound (ABB); (**I**) The test for presence of dihydroxyphenols of a swollen mound with no detection (FeCl_3_). *ab*—abaxial (outer) side; *e*—epidermis; *ie*—inner epidermis, inside the saccate lip base; *r*—idioblast with raphide crystals; *se*—subepidermis; *sm*—a swollen mound; *vb*—vascular bundle.

**Figure 5:**
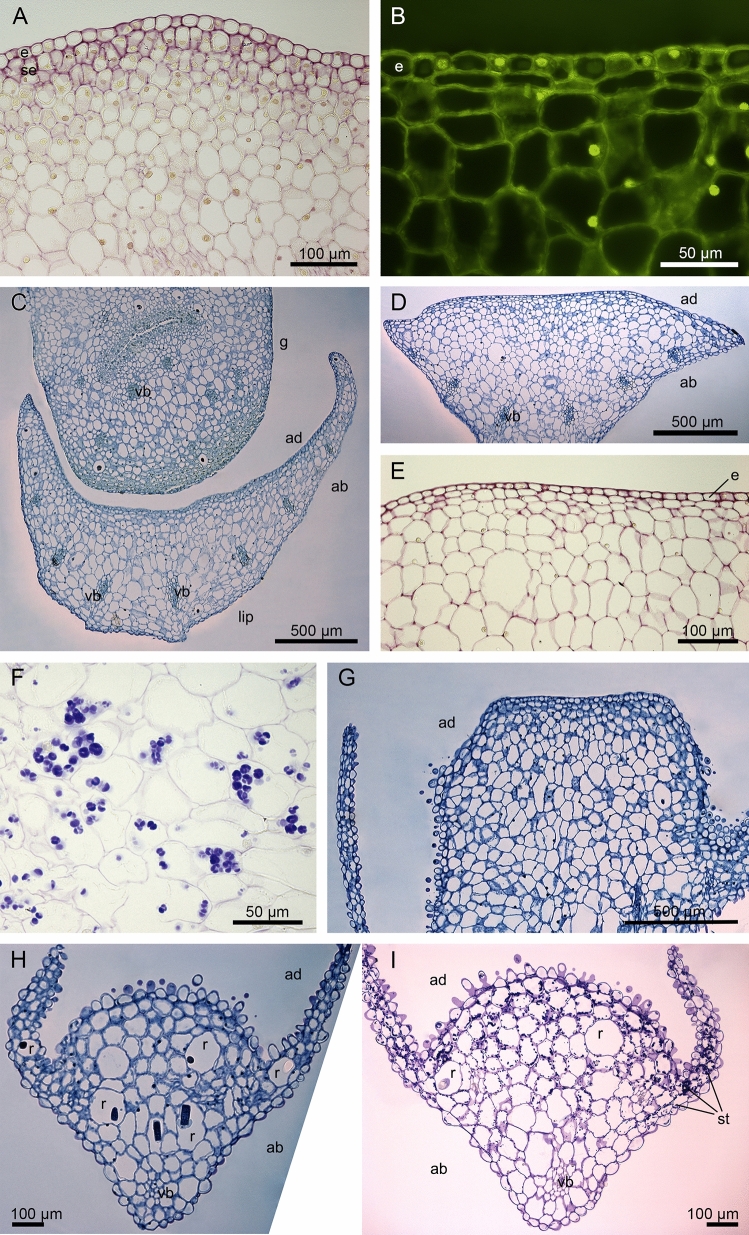
Histochemical tests of lip and the swollen mound of gynostemium: (**A**) The transverse section of the swollen mound with no detection of pectic acids/mucilage (Ruthenium Red); (**B**) The middle part of the lip with an unruptured cuticle on epidermal cells (Auramine O); (**C**, **D**) The small cells of epidermis and subepidermis with dense cytoplasm of gynostemium, but rather not active lip cells (TBO); (**E**) No detection of pectic acids/mucilage of the middle part of the lip (Ruthenium Red); (**F**) Few starch grains in ground parenchyma; (**G**) The part of the lip before the apex with papillate margins, noticeable slight outline of secretory activity (TBO). (**H**, **I**) The papillate apex with many idioblasts cells with raphide crystals (TBO) and starch grains (PAS). *ab*—abaxial (outer) side; *ad*—adaxial (inner) side; *e*—epidermis; *g—*gynostemium; *r*—idioblast with raphide crystals; *se*—subepidermis; *st*—starch grains; *vb*—vascular bundle.

**Figure 6:**
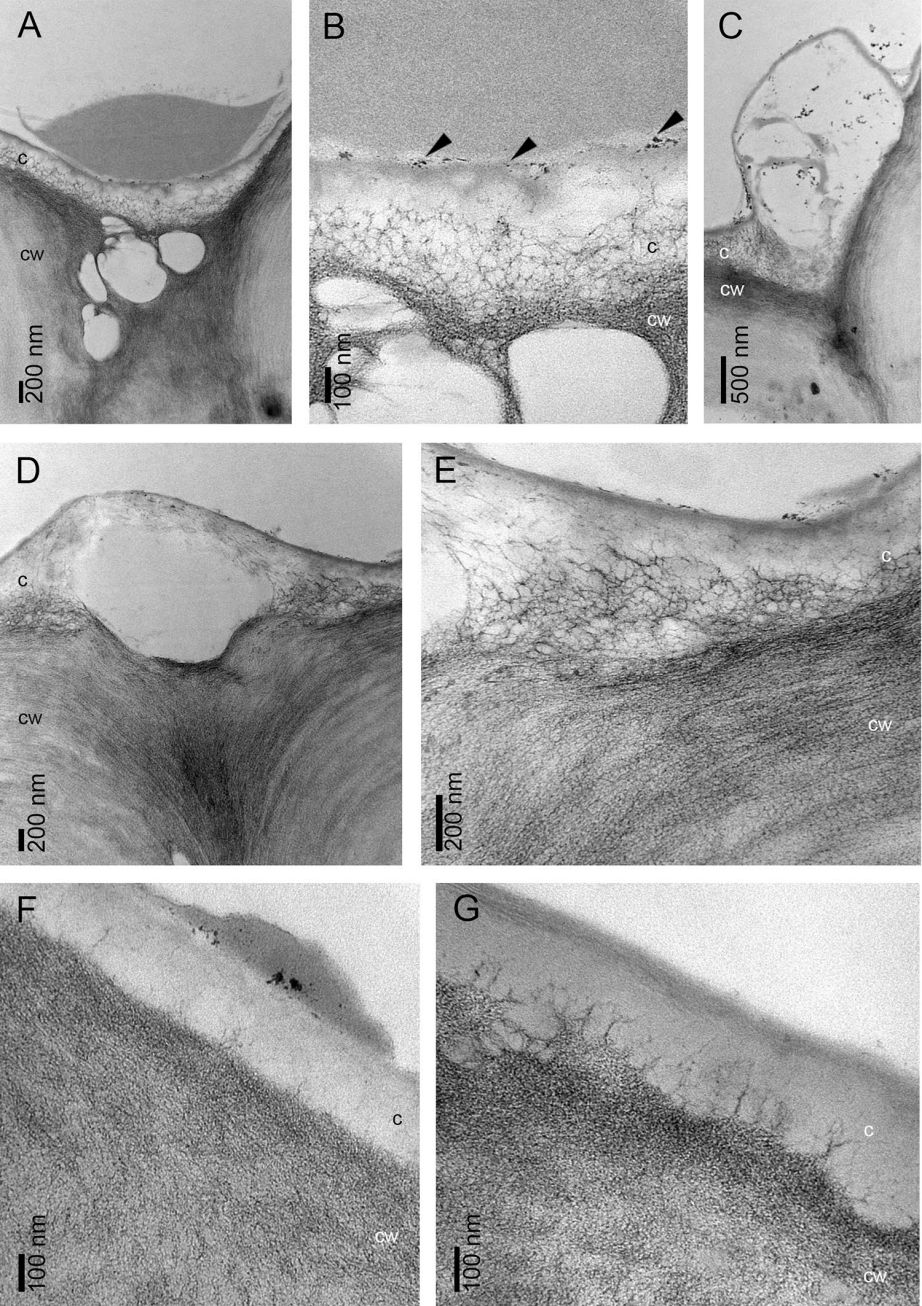
Lip base in the bud: (**A**) The residues of secreted material on the cuticle surface and between radial cell walls (TEM); (**B**) The dense net of micro-channels visible in the cuticle and the remnants of exuded substances on its surface (*arrows*) (TEM); (**C**) Cuticle swellings caused by accumulation of secretory products beneath (TEM); tangential cell wall thickness between 1,1-1,6 µm; Middle part of lip in the bud: (**D**) Thick tangential cell wall range between 2,3-2,9 µm; (**E**) the residues of secretions accumulated beneath the cuticle passing through micro-channels; Labellar margin: (**F**) slight amount of secretions on the cuticle; (**G**) Micro-channels in the cuticle. *c*—cuticle; *cw*—cell wall.

The transverse sections from saccate lip base and the swollen mound reveal a single layer of epidermis and a few subepidermal layers with cells with dense cytoplasm, only exclusively in the swollen mound of gynostemium (Fig. 4A–C). The swellings are noticeable on epidermal cells of the mound (Fig. 4D). The cells of ground parenchyma are vacuolized and among them collateral vascular bundles occur (Fig. 4A–E). The idioblasts contain bundles of raphides sharp needle-like crystals of calcium oxalate (Fig. 4E). The starch grains, detected in the PAS method, occur in deeper layers of parenchyma, not in epidermis (Fig. 4F, G). The other tests: ABB for proteins, FeCl_3_ for dihydroxyphenols, Ruthenium Red for pectic acids/mucilage do not detect these components (Figs. 4H, I, 5A). The fluorescence staining with Auramine O displays unruptured cuticle on epidermal cells (Fig. 5B), which is also visible in TEM results (compare with Fig. 6D, F). The further lip and gynostemium sections, above the mound, reveal the small cells of epidermis and subepidermis with dense cytoplasm of gynostemium, but rather not active lip cells (Fig. 5C–E). No detection of pectic acids/mucilage of the middle part of the lip was observed (Ruthenium Red; Fig. 5E). In ground parenchyma few starch grains are still noticeable (Fig. 5F). The shape of lip changes towards the apex into papillate and slight outline of secretory activity are noticeable (Fig. 5G, compared with Fig. 3D, E). The papillate apex contains many idioblasts cells with raphide crystals and starch grains (Fig. 5H, I). The set of different histochemical tests revealed the localization of substances through floral tissue and allowed us to compare these results with the chemical composition obtained from the GC–MS method.

TEM observations show the residues of secreted material on the cuticle surface and between radial cell walls (Fig. 6A). The dense net of micro-channels is visible in the cuticle with the remnants of exuded substances on its surface (Fig. 6B) and cuticle swellings caused by accumulation of secretory products beneath (Fig. 6C). The secretory material on the surface is heterogenous (compare Fig. 6A, C). The residues of secretions in the middle part of the lip accumulate beneath the cuticle (Fig. 6D) passing through micro-channels (Fig. 6E). Slight amounts of secretions on the cuticle of lip margins near the apex (Fig. 6F) are transported via micro-channels (Fig. 6G).

**Figure 7:**
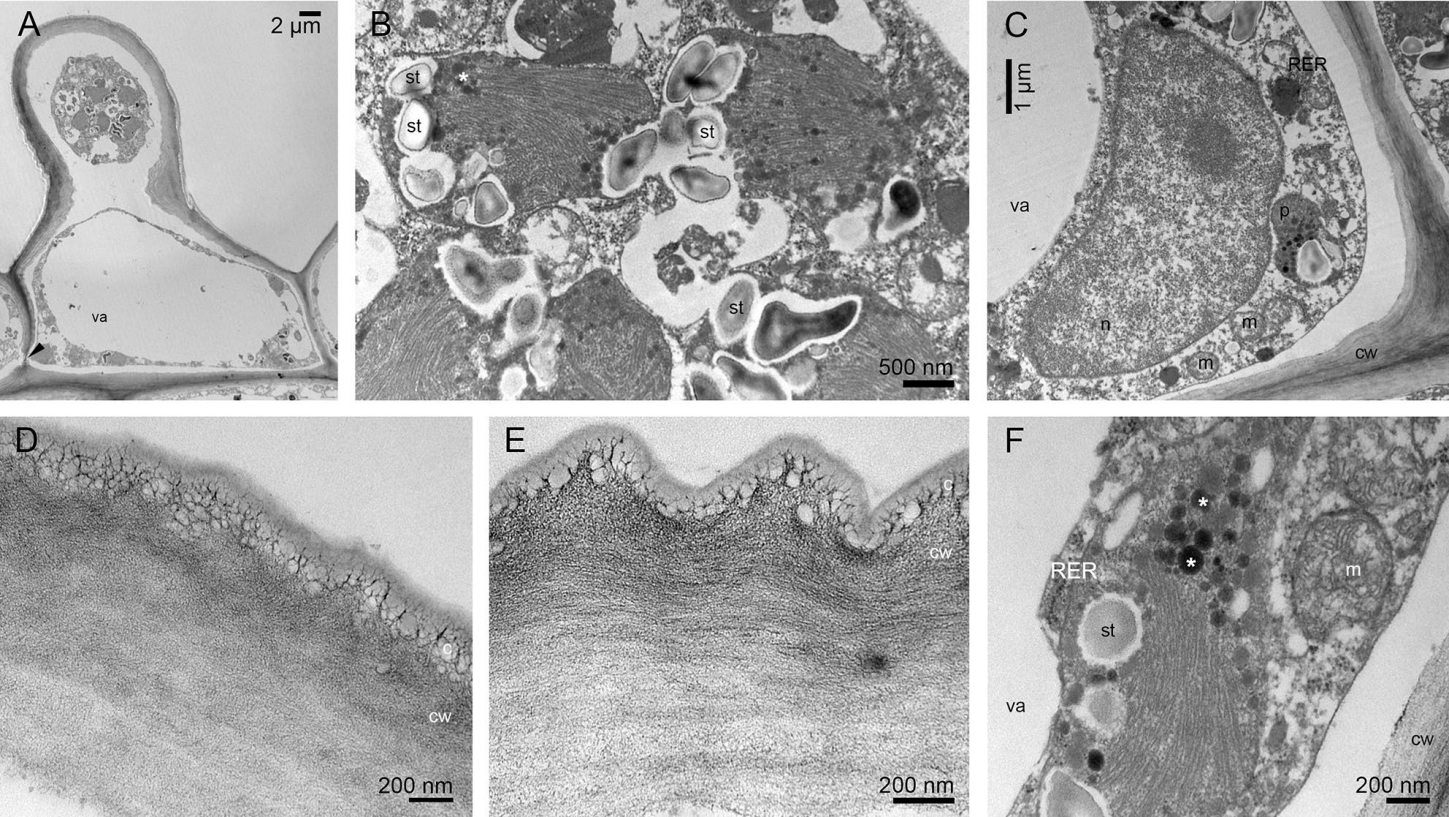
The bud: (**A**) Obpyriform papilla (TEM); (**B**) Details of (**A**) The apex of a papilla with plastids equipped with lamellae, starch grains and plastoglobuli; plasmodesmata (*arrow*) present in the cell wall (TEM); **C.** Details of (**A**) The bottom of the papilla with large nucleus, plastids, mitochondria, RER profiles (TEM); (**D**) Micro-channels visible in the cuticle of papillae (TEM); (**E**) Striate cuticle with reticulation on flat cells near the papilla, note remnants of secreted materials on cuticle (TEM); (**F**) Mitochondria, plastids with starch grains and plastoglobuli (*asterisks*), RER profiles in the flat cell (TEM). *c*—cuticle; *cw*—cell wall; *m*—mitochondrion; *n*—nucleus; *p*—plastid; *RER*—rough endoplasmic reticulum; *st*—starch grains; *va*—vacuole.

TEM observations of obpyriform papillae (Fig. 7A) show cytoplasm containing plastids with starch grains, plastoglobuli and lamellae (Fig. 7B), large nucleus (Fig. 7C). The cuticle reticulation (micro-channels) is clearly visible in the cuticle of papillae (Fig. 7D) and also in flat cells (Fig. 7E), and some remnants of secreted materials are marked on the surface. Mitochondria and RER profiles are shown (Fig. 7F). The results from ultrastructural observations allowed us to draw conclusions about the secretory process.

### Nectar analysis

Nectar is strongly sucrose-dominant, with an average contribution of sucrose as high as 93% of the total carbohydrates (Table 1). Fructose and glucose are both much more abundant in methanolic extract from the flower tissue, where they constituted ca. 50% of all sugars, suggesting that extraction procedure allowed to separate the nectar itself. No volatiles were detected in dichloromethane extracts.

**Table 1:** The relative composition (% weight) of the carbohydrate fraction of nectar (*n* = 4, mean ± SD) and sugars extracted from the whole floral tissues.

Compound	Nectar (%)	Extract from tissues (%)
Fructose	4.2 ± 3.3	26.4
Glucose	2.6 ± 1.4	22.7
Sucrose	93.2 ± 4.6	51.0

## Discussion

Hummingbirds caught in action transferring the pollinia on their beaks and the presence of fruit set after their visits proved the hypothesis that *O. fulgens* (Maxillarinae) is bird-pollinated. These observations with morphological and anatomical studies give the proof after 40 years of seeking the evidence of bird pollination within representatives of Maxillarinae. Observations of such type were previously documented for other members of the orchid family, such as *Stenorrhynchos lanceolatus* (Aublet) L. C. Rich. (Spiranthinae) which has been pollinated by *Phaethornis eurynome* (Phaethorninae), *Thalurania glaucopis* (females only) and *Leucochloris albicollis* (both belonging to Trochilinae)^26^. However, the presented paper concentrates on morphological and anatomical features of bird pollination syndrome, or in other words the plant’s adaptation to ornithophily, which embraces several features in floral morphology that have evolved to facilitate it. The identification of floral features of the bird syndrome, with later evidence of bird pollination, is a huge step toward interpretation of flower-pollinator interactions and can influence both species' protection. Flowers of *O. fulgens* meet practically all of the criteria of bird-pollination syndrome. The most obvious one is the vivid coloring and contrasting lip, in this case tepals are intensive red with yellow to orangish lip. Red coloration is thought to play a major role in both bee-deterrence (making them invisible for bees) and bird-attraction (being readily detectable)^1^. However, it should be noted that bees can perceive some flowers seen as red by humans, if they have at least some reflectance in the shorter wavelengths as well^27^. There is an ongoing discussion regarding the sense of smell among different bird groups and whether they use, and if yes to what extend, while searching for food (e.g.,^28–31^). There is, however, very little information about the role of smell in foraging by nectarivorous birds^32^, and since the purpose of the presented work was not to investigate this issue, we will follow the widely held notion that the sense of smell is not dominant in birds thus bird pollinated flowers are usually lacking any detectable scent. Nevertheless, it should be noted that the latest work published by Núñez et al.^32^ proved that *Amazilia amazilia s. l.*, species closely related to the one pollinating scentless *O. fulgens*, does not use smell in the search for food but employ other senses, mainly sight. During the course of this research, we have investigated this aspect, however, we have not found any volatile compounds. This result was also supported with our field observations. *O. fulgens* has pendant, campanulate flowers with labellar margins curved back, weakly expressed zygomorphy and diurnal anthesis. Floral tissues are clearly strengthened and rigid, giving the impression of being crispy and thus they can withstand contact with a hard beak. In ornithophilous flowers the nectar guides are absent^2^, and the investigated species is no exception. Similar floral morphology has been previously described e.g. in closely related, and also possibly bird-pollinated, *O. coccineum* (= *M. coccinea*), which as well has weakly zygomorphic, globose flowers with scarlet tepals and a scarlet-yellow backwardly curved lip and diurnal anthesis. It also produces abundant nectar and lacks nectar guides. In *O. fulgens,* however, the lip is essentially unlobed, and without callus, whereas in *O. coccineum* it is 3-lobed, with a simple, hemispherical callus. Other examined orchid species, presumably ornithophilous, also possess vivid flower colours: *Hexisea imbricata* (Lindl.) Rchb. f. (Laeliinae) has scarlet perianth with a distinct yellow labellar callus^33^, *Symphyglossum sanguineum* (Rchb. f.) Schltr. (Oncidinae) has pink-violet flowers with labellar auricles^34^, and *Comparettia falcata* Poepp. & Endl. has reddish or pink flowers with labellum which ends in two extensions (horns) and extend longitudinally within the spur^35^. In the aforementioned species, the lip is saccate, rigidly attached to the column and floral tissues are tough. Furthermore, in *O. coccineum* a strong fold in the lip partially closes the floral tube at the level of the anther and stigma and thus probably forces the bird to push its beak against the column to gain entry^2^. As already mentioned, ornithophilous flowers tend to lack odor, however, in *O. coccineum* sweet honey-like scent was occasionally detected^23^, which is a significant difference between this species and *O. fulgens.*

Pollinia in bird-pollinated Orchidaceae are often brown, as Dressler^36^ has pointed out, whereas those of their insect pollinated relatives are yellow. However, it seems that exceptions to these rules are common. In his paper it is indicated that many other orchids, which show the syndrome characteristic of hummingbird pollination, have creamy or greyish white pollinia (in contrast to the most common bright yellow, characteristic for insect pollination). As examples, Dressler has mentioned among others *H. imbricata*, *S. sanguineum*, *C. falcata* and *Maxillaria fulgens* (= *O. fulgens*). Indeed, this is the only characteristic for bird-pollinated orchid flowers that is lacking in investigated *O. fulgens*, which similarly to most (if not all) Maxillariinae representatives, has creamyyellowish pollinia. The very same phenomenon is described by Catling^37^ for *Sacoila lanceolata*, in which hummingbird pollination has also been reported by the author.

The nectaries in *O. coccineum* and *H. imbricata* are characterized by a pronounced protuberance at the base of the column and nectar gathers in the reservoir formed by the fused lip and the base of the column-foot, like “faucet and sink” arrangement. Such protuberances are known also from floral desiccations of *O. fulgens* (= *M. fulgens*) and other *Ornithidium* representatives, also entomophilous, e.g*.* in *O. parviflorum* (Poepp. & Endl.) Rchb. f. (= *Maxillaria parviflora* (Poepp. & Endl.) Garay), *O. aggregatum* (Kunth) Rchb. f. (= *M. aggregata* (H.B.K.) Lindl.), *O. nubigenum* Rchb. f. (= *M. nubigena* (Rchb. f.) C. Schweinf.), *O. ruberrimum* (Lindl.) Rchb. f. (= *M. ruberrima* (Lindl.) Garay), and *O. sophronitis* Rchb. f. (= *M. sophronitis* (Rchb. f.) Garay (^23^ and references therein). The exact function of this protuberance remains unclear, as it has been interpreted as a non-secretory *tabula infrastigmatica*^38^, e.g. in *O. parviflorum* where nectar is secreted at the lip surface inside the cavity and offered there (the Singer’s proposal about “faucet and sink” arrangement) or as a nectary, e.g. in *O. coccineum*^23^ and *H. imbricata*^33^. In *O. fulgens*, the epidermis of the basal protuberance of column-foot has features advocating for secretory activity, which confirms Singer's hypothesis of „faucet and sink”. A small amount of secretions are found in the middle part of the lip, which is puzzling. In the papers of Stpiczyńska et al.^23,33^, the authors did not include any information regarding nectaries located on the lip, thus it may be assumed that they may be found also in *O. coccineum* and *H. imbricata.* Histochemical and TEM research of other *Ornithidium* species may clear up the mystery.

In *O. fulgens*, the nectary consists of a single-layered epidermis with smaller cells and few layers of subepidermis. Larger and more vacuolised parenchyma cells with few collateral vascular bundles occur beneath them. This pattern of floral nectariferous tissue is often described in orchids, i.e. in presumably ornithophilous: *O. coccineum*^23^, *O. sophronitis*^24^, *H. imbricata*^33^, and *S. sanguineum*^34^, but also in some sapromiophilous representatives of the genus *Bulbophyllum* Thouars^39,40^ as well as entomophilous species of *Epipactis* Zinn^41,42^ and *Neottia* Guett.^43^ or also in some other species^44^. In *O. fulgens*, unlike in *O. coccineum*^23^, the secretory cells do not possess very thick walls. In both species, however, flowers are rather stiff and crispy in touch, which may be an adaptation to prevent mechanical damages caused by the hummingbirds’ beaks^23^. Like in *O. coccineum*^23^ and *H. imbricata* ^33^, SEM and histochemical studies in *O. fulgens* did not demonstrate any pores or cracks the paths for nectar release. In all these species, the cuticle has characteristic swellings. However, the difference in its sizes is significant: in *O. fulgens* the swellings were up to 1.6 µm high, in *O. coccineum*: 2 ± 7 mm high, and in *H. imbricata*: ± 2 µm high. Furthermore, in both species of *Ornithidium* they are formed at points coinciding with the middle lamella between adjoining epidermal cells. In *O. coccineum* the swellings occured only on the surface of the column-foot protuberance (nectary), whereas in *O. fulgens* they were also present on the surface of the lip base. In both mentioned species they were absent from the epidermal cells of the nectar reservoir. Moreover, in both taxa, the abundant nectar filled the space between the lip and gynostemium, like in the container^34^. The cuticular swellings and uninterrupted layer of reticulate cuticle on the tangential walls of the secretory epidermis suggest that nectar accumulation is taking place beneath the cuticle causing its stretching and then secretion *via* micro-channels to the exterior. The reticulate cuticle with micro-channels is a frequent phenomenon in orchid nectaries and osmophores^33,39,40^.

The secretory epidermal cells of *O. fulgens* contained plastids with starch grains (amyloplasts), similarly as in *H. imbricata*^33^ and *S. sanguineum*^34^, however not present in *O. coccineum*^23^. The starch grains commonly occur in plastids of nectariferous cells^39,40,44–47^. Starch is utilised as a source of sugar and energy for metabolic processes during nectar secretion^48^, so the presence/absence as well as number and volume of amyloplasts can describe the suitable stadium of anthesis: pre- or post-secretory^24,39,44^. At the highest level of nectar secretion in *H. imbricata* the plastids contained both starch grains and plastoglobuli. During starch depletion, the plastids became elongated, irregular in shape and more plastoglobuli (described as lipid droplets within the plastids) occurred. In cytoplasm, the lipid droplets (sometimes described as osmiophilic content) were accumulated^33,34,39,40,45^. They also were present in *O. fulgens*. Lipids are sometimes considered to be the counterparts of fragrance^49–51^. The observed profiles of endoplasmic reticulum, few vesicles close to plasmalemma, micro-channels in cuticle, and cuticular swellings suggest the granulocrine route of nectar release, the same as in *H. imbricata*, where arrays of ER and dictyosomes were participating in nectar secretion. Also, the vesicles were visible in cytoplasm and near the plasmalemma. After crossing the outer tangential walls of nectariferous epidermis, the released substances accumulate beneath the cuticle and exceed the cuticle forming micro-channels or by rupturing it. We did not observe the cracked cuticle, only the swellings and the cuticle reticulation, but both ways of nectar release are possible.

Idioblasts with raphides of calcium oxalate crystals surrounded by mucilage in subepidermis and parenchyma cells that were observed in *O. fulgens,* are thought to deter herbivores, and has been previously frequently reported in orchids (i.e.^39,42,52^. Mucilage in idioblasts was also present in *H. imbricata*^33^. Davies has reported the presence of raphides in leaf and floral tissues^53^ for a number of *Maxillaria* spp. and has also suggested that they may be secretory products and may perhaps discourage herbivory by invertebrates^53,54^.

The main role of nectar is the attraction of potential pollinators. In the past it has been considered to be a simple sugar solution, however it is now known that it consists of a variety of chemicals dissolved, or suspended, in an aqueous solution^55^. These may range from mixtures of one to three common sugars, such as glucose, sucrose and fructose, to more complex sugar solutions^56^ or combinations of sugars, free amino acids, vitamins, lipids, and other compounds^57,58^.

The chemical analysis conducted by us proved that the liquid collected from the flowers of *O. fulgens* is a nectar, with sucrose as a dominant constituent. Data presented by Baker et al.^59^ strongly supported the hypothesis that the composition of soluble sugars in nectar is influenced by the pollinators that consume it. Nectars of flowers visited by hummingbirds or Megachiroptera tended to have high levels of sucrose^59–61^ while nectars of flowers consumed by passerines had very low levels of this sugar^59^. Bee-pollinated flowers also have sucrose-rich or sucrose-dominant nectar^62^, so such nectar composition in hummingbird-pollinated flowers that have evolved from bee-pollinated flowers is rather not surprising^1^. Similar conclusions were published by several other researchers^63–67^. Nectar produced by closely related *O. sophronitis* has been tested using refractometry (concentrations) and glucose-sensitive test sticks (Clinistix) and it has presented the value of 64% (w/w) sugar and the presence of glucose has been confirmed^24^. In *Maxillaria anceps* Ames & C. Schweinf., presumably bee-pollinated species with strongly zygomorphic, relatively open, greenish-white flowers with a well-developed lip, nectar is also sucrose-dominant, but contains low concentrations of glucose, fructose, free amino acids and possibly terpenoids^68^. In our study, nectar was strongly sucrose-dominant, with an average contribution of sucrose as high as 93% of the total carbohydrates. This together with morphological evidence itself advocates strongly in favor of ornithophily as the pollination syndrome in *O. fulgens.*

## Conclusion

The field observations of regular visits of the azure-crowned hummingbirds (*Amazilia cyanocephala*) hovering and transmitting the pollinia of *Ornithidium fulgens*, as well as floral morphology and anatomy undoubtedly prove that this species is bird-pollinated and thus that such syndrome occurs within the members of the subtribe Maxillariinae Benth. The next steps in our research will be to study the continuation of the hummingbirds' visits to the flowers and their participation in the fruit set (with statistics).

## Materials and methods

Plants of *O. fulgens* have been cultivated in the Estación Experimental de Orquídeas de la Familia Archila (Cobán, Guatemala), the seminatural plantation located in a cloud forest of Guatemala. The flowering season *in situ* ranges mostly from September to January, with the peak season in November and December. Tissue samples were collected from fresh flowers at different stages of anthesis.

Morphological analysis has been conducted with methods of classical taxonomy. Formal identification of the plant material has been performed in Guatemala by Fredy L. Archila Morales and Monika M. Lipińska. Voucher specimen has been deposited in BIGU herbarium. Research complied with relevant institutional, national, and international guidelines and legislation.

Samples for the scanning electron microscopy (SEM) were preserved in 2.5% (*v*/*v*), glutaraldehyde (GA) in 0,05M cacodylate buffer (pH 7,0). Following dehydration in an ethanol series, they were dried by the critical point method using liquid CO_2_, and coated with gold and observed by means of a Philips XL-30 scanning electron microscope.

For histochemical studies, the plant material was fixed in 2.5% (*v*/*v*), glutaraldehyde (GA) in 0.05 M cacodylate buffer (pH = 7.0) was used. Then the material was rinsed with cacodylate buffer and dehydrated in the ethanol series. Finally, the tissue fragments were embedded in methylmethacrylate-based resin (Technovit 7100, Heraeus Kulzer GmbH). Sections were cut with glass knives (5–7 μm thick) using a Leica EM UC 7 ultramicrotome and mounted on glass slides. The semi-thin control sections were stained with 0.05% (*w*/*v*) aqueous Toluidine Blue O (TBO, C.I. 52040)^69,70^. The detection of water-insoluble proteins was possible with the test of Aniline Blue Black (ABB, C.I. 20470)^71^. The water-insoluble polysaccharides, especially starch grains, were detected in the Periodic Acid-Schiff reaction (PAS)^71^. The pectic acids/mucilage were identified following test with a 0.05% (*w*/*v*) aqueous Ruthenium Red (C.I. 77800) solution^72^, whereas catechol-type dihydroxyphenols following staining with a 10% (*w*/*v*) aqueous solution of FeCl_3_^73^. The preparations were studied and photographed with a Nikon Eclipse E 800 light microscope and a Nikon DS-5 Mc camera using Lucia Image software. The sections, following FeCl_3_ test, were examined using the differential interference contrast (DIC) imaging. Auramine O (C.I. 41000) 0.01% (*w*/*v*) solution in 0.05 M buffer Tris/HCl, pH = 7.2 was used to detect the presence of cuticle^74^, particularly unsaturated cutin precursors and acidic waxes^73^ and tissue slides were examined with a Nikon Eclipse E800 fluorescence microscope, equipped with filter B-2A (EX 450–490 nm, DM 505 nm, BA 520 nm).

For transmission electron microscopy (TEM), the lip was fixed in 2.5% (*v*/*v*), glutaraldehyde (GA) in 0.05 M cacodylate buffer (pH 7.0). Post fixation overnight in 1% OsO_4_ in the cacodylate buffer. The samples were dehydrated by means of the graded acetone series and embedded in Spurr’s resin. Ultrathin sections (60 nm) were cut using a Leica UC7 ultramicrotome. Sections were examined by means of a FEI Tecnai Spirit BioTWIN transmission electron microscope at 120 kV.

For chemical analyses, nectar secreted by ca. 15 flowers during the first day of anthesis, was carefully collected using several small pads of glass wool, which were then extracted in 10 ml methanol. Whole flowers were subjected to sequential organic solvent extraction. First, non-polar compounds were isolated in 10 ml dichloromethane for 20 s, then carbohydrates were extracted by dipping flowers for 30 s in 10 ml methanol. Extracts were then stored at 4 °C prior to analysis. Due to the difficult conditions of the nectar collection in the field we were unable to reliably determine its volume and as a consequence the nectar concentration remains unknown.

The dichloromethane extract was concentrated to ca. 0.3 ml under a stream of nitrogen. Samples were then analyzed using gas chromatography mass spectrometry (GC-MS), which was performed using a Shimadzu QP-2010SE system (Shimadzu, Kyoto, Japan), equipped with a 30 × 0.25 mm i.d., film thickness 0.25 μm, ZB-5ms capillary column (Phenomenex, Torrance, CA, USA). Helium was used as the carrier gas at a flow rate of 1 ml min^−1^. The split ratio was 1:10, and the injection volume was 1 μl. The injector and GC-MS interface temperatures were maintained at 310 °C. Electron ionization (electron energy 70 eV, ion source temperature 200 °C) was used. The column temperature was programmed for 30 °C (isothermal for 3 min) to 180 °C at 4 °C min^−1^, then from 180 to 310 °C at 8 °C min^−1^, and then maintained at 310 °C for 12 min.

Carbohydrates present in methanolic extracts were subjected to sequential derivatization procedure according to the slightly modified method described by Ruiz-Matute et al.^75^. An aliquot of each extract was evaporated to dryness under a stream of nitrogen. Then, oximes were synthesized by adding 0.1 mL of a 2.5% hydroxylamine hydrochloride solution in pyridine. Oximes obtained this way were transferred to respective trimethylsilyl (TMSi) derivatives by adding 0.1 mL BSTFA + TMCS (99:1). Each reaction was performed at 70 °C for 30 min. Derivatives were analyzed using gas chromatography with a flame ionization detector (GC-FID). The analysis was performed using a Clarus 500 gas chromatograph (Perkin-Elmer Instruments, Waltham, MA, USA), equipped with the same type of column as mentioned above. The column temperature was programmed from 80 to 300 °C at 4 °C min^−1^. Injector and detector temperatures were set at 320 °C. Argon was used as carrier gas at a flow rate of 1 mL min^-1^. The split ratio was 1:20, and the injection volume was 1 μL. Identification was based on retention times, which were compared to those of analytical standards of glucose, fructose and sucrose analyzed in the same conditions.
